# Work-Related Psychological Injury Is Associated with Metabolic Syndrome Components in Apparently Healthy Workers

**DOI:** 10.1371/journal.pone.0130944

**Published:** 2015-06-18

**Authors:** Nicola Magnavita

**Affiliations:** Department of Public Health, Università Cattolica del Sacro Cuore, Roma, Italy; Max Planck Institute of Psychiatry, GERMANY

## Abstract

**Objective:**

The aim of this study was to evaluate the association between psychological damage caused by common occupational trauma and metabolic syndrome (MES).

**Method:**

571 workers from 20 small Italian companies were invited to fill in the Psychological Injury Risk Indicator (PIRI) during their routine medical examination at the workplace.

**Results:**

Compared to workers with no psychological injury, workers with a high PIRI score had a significantly increased risk of having at least one metabolic syndrome component (adjusted hazards ratio, 1.8; 95% confidence interval, 1.2 to 2.6). There was a significant increase in the risk of hypertriglyceridemia in male workers (OR 2.53 CI95% 1.03-6.22), and of hypertension in female workers (OR 2.45 CI95% 1.29-4.66).

**Conclusion:**

Psychological injury related to common occupational trauma may be a modifiable risk factor for metabolic syndrome.

## Introduction

Metabolic syndrome (MES) is a recently identified disorder involving a cluster of risk factors that may include abdominal obesity, dyslipidemia, reduced glucose tolerance and hypertension. The incidence of this syndrome is already high and is increasing in most parts of the world [1]. The etiology of MES is clinically highly relevant since it can lead to life-threatening complications such as ischemic heart disease and other cardiovascular diseases, diabetes, kidney failure, and certain types of cancer. Contributing factors include a sedentary life-style combined with increased dietary fat intake as well as psychosocial factors. The term "psychosocial factors" is extremely vague and covers a wide spectrum of conditions ranging from the presence of mental illness, exposure to trauma in everyday life, chronic exposure to work stress, and others.

Psychological disorders such as depression and anxiety have consistently been reported to be associated with MES [2–4]). In both a Finnish study on the general population [5] and a Japanese research report [6], psychological distress, as measured by the General Health Questionnaire, significantly increased the risk of MES. Exposure to major life events [7] and marital dissatisfaction also proved to be relevant risk factors for MES [8–10]. Occupational and non-occupational trauma inducing post-traumatic stress disorder (PTSD) may also predispose to MES and to cardiovascular diseases [11–12]. Work-related stress has been reported to be associated with MES [13–14]), though the causal relationship between the two remains unclear. It is difficult to make a review of published studies as the variable selected and study designs are heterogeneous [15]. However, there remains the legitimate question of whether a trauma can cause occupational MES. While a large body of literature indicates an association between PTSD and MES in the presence of major traumatic events [11,12],the real issue is whether small traumas that anyone can suffer at work may be associated with an increase in MES or its components.

The fifth Diagnostic and Statistical Manual of Mental Disorders (DSM-5), issued in May 2013, introduced changes to the diagnostic criteria for PTSD and Acute Stress Disorder, taking into account new aspects arising from scientific research and clinical experience. A new class of "trauma and stressor-related disorders" includes as a diagnostic criterion conditions that require exposure to a traumatic or stressful event. The rationale underlying the creation of this new class was based upon clinical recognition of a number of expressions of distress resulting from traumatic experience. It is well known that some jobs such as law enforcement, firefighting, military activities and other first-response occupations, expose workers to high intensity trauma that can cause PTSD, but psychological injury may also be associated with work-related stress in ordinary occupations that do not require immediate action. Indeed, severe incidents occur with a relatively low frequency, while in many jobs, the prevalence and frequency of minor occupational trauma such as being exposed to physical or verbal violence, being directly involved in/ witnessing an accident or collecting unforeseen negative experiences are high.

Since studies in the literature on trauma and MES have been carried out mainly on clinical cases (i.e. people with psychiatric illness or PTSD) or on first-response occupations, our aim was to determine whether the relationship between occupational stress, psychological injury and MES was also evident in a sample of apparently healthy workers exposed to everyday occupational events.

## Methods

### Subjects and ethics statement

Italian law requires workers to undergo regular health checks at the workplace. Workers from 20 small companies involved in different economic sectors were asked to complete a questionnaire before their routine medical examination. All healthy workers were eligible for the study. Since health surveillance is performed in the workplace, workers who had any physical or mental illness (including trauma and stress-related disorders) causing sickness absence could not be examined. Participation in the research was not mandatory, but almost all workers agreed to participate (571 out of 577, participation rate 99%). Participants provided their written informed consent to participate in this study by signing a form in their own personal health document. The Ethics Committee of the Università Cattolica del Sacro Cuore approved the study design and the consent procedure. The characteristics of the sample are reported in Table 1.

**Table 1:** Population and data.

	N	%
**Total**	571	100.0
Gender: Male	188	32.9
Female	383	67.1
Smoker	214	37.5
Alcohol use	49	8.6
Lack of physical activity	388	68.0
Lack of sleep	71	12.4
**Metabolic syndrome components**		
Reduced HDL cholesterol (HDL-c)	23	4.0
Elevated triglyceride (TGs)	57	10.0
High blood pressure (BP)	66	11.6
High fasting plasma glucose (FPG)	42	7.4
Central obesity	68	11.9
At least one MS component	182	31.9
Metabolic syndrome	10	1.8
	**mean**	**s.d.**
Age, years	44.2	10.2
PIRI standardized score (range 0–100)	17.5	15.4
	**N**	**%**
Workers with PIRI score >25	139	24.3
MES in workers with PIRI>25	6	4.3
Reduced HDL-c in workers with PIRI>25	7	5.0
High TGs in workers with PIRI>25	21	15.1
High BP in workers with PIRI>25	27	19.4
High FPG in workers with PIRI>25	14	10.1
Obesity in workers with PIRI>25	18	12.9

### Questionnaire

The questionnaire collected information on gender, age, smoking status (current smoker, nonsmoker), physical activity (regular/none), alcohol intake (less than 7 alcohol units per week/ 8 or more alcohol units per week), sleep habit (regular/disturbed), and included the Psychological Injury Risk Indicator (PIRI), a self-report measurement specifically developed to enable the early identification of evolving psychological injury among workers [16]. The PIRI scale, in its Italian version [17], proved to be a valid tool for providing accurate routine psychological health assessment, and seemed to be a significant indicator of traumatic experiences in the workplace. The PIRI contains 26 items, with answers graded on a Likert (0–6) point scale, comprising 4 sub-scales: A “sleep problems”(6 items), B “recovery failure” (5 items), C “post-traumatic stress symptoms” (10 items), and D “chronic fatigue” (5 items). The Cronbach α coefficient of internal consistency for the overall PIRI questionnaire was calculated to be 0.93. The original PIRI version also contains 4 dichotomous (yes/no) items, corresponding to the Rapid Alcohol Problems Screen (RAPS 4) measure, a four-question quiz designed for detecting alcohol dependence [18]. Since there was a very low frequency of positive answers for these items, they were not used in this sample. The total score based on the sum of the PIRI Likert scores was transferred to a 0–100 scale. A standardized PIRI score of over 25 corresponded to potential psychological injury, while higher scores indicated a greater risk of injury [19].

### Biologic data

In addition to the self-reported information, subjects also provided biologic data. Subject height and weight were recorded, and the waist circumference was obtained by measuring the narrowest point between the lowest costal (tenth rib) and the iliac crest. BMI was calculated as weight (kg) divided by square height (m2). Fasting blood glucose (FBG) and blood lipid profile (total cholesterol, high-density lipoprotein, triglycerides, and low-density lipoprotein) were determined.

According to the International Diabetes Federation (IDF) guide [20], and the National Cholesterol Education Program Expert Panel on Detection Evaluation and Treatment of High Cholesterol in Adults (NCEP/ATPIII) [21] the following criteria are an indication of the presence of metabolic syndrome: central obesity (defined as BMI>30 kg/m^2^, or increased waist circumference with ethnicity-specific values); elevated triglyceride (TGs) level: > 150 mg/dL (1.7 mmol/L), or specific treatment for this lipid abnormality; reduced HDL cholesterol (HDL-c): < 40 mg/dL (1.03 mmol/L) in males, < 50 mg/dL (1.29 mmol/L) in females, or specific treatment for this lipid abnormality; high blood pressure (BP): systolic BP > 130 or diastolic BP >85 mm Hg, or treatment of previously diagnosed hypertension; high fasting plasma glucose (FPG): >100 mg/dL (5.6 mmol/L), or previously diagnosed type 2 diabetes. According to NCEP/ATPIII MES [21] diagnosis requires the presence of three or more of the above components.

All blood measurements were carried out in an accredited laboratory with automatic biochemistry coulter analyzer using standardized immunoenzymatic methods; total cholesterol, HDL-c and TGs methods meet the NCEP criteria for precision, accuracy, and total error [22].

### Statistics

Chi-square tests were used to compare prevalence among subgroups defined by psychological dimensions and gender. Fisher's exact test was used in the analysis of contingency tables when sample sizes were small. Student’s t test was used to compare subgroups means defined by job type. Linear regression was used to study the association between psychological injury (standardized PIRI score) and the number of MES components.

Univariate logistic regression analysis was used to study the association between potential psychological injury (high PIRI score) and the presence of at least one metabolic syndrome components (obesity, hypertension, dyslipidemia, diabetes). Independent variables (age, sex, smoking, alcohol use, lack of physical exercise, sleep deprivation) were carried forward to multivariable binary logistic regression for adjustment. Logistic regression analysis was also carried out to evaluate the association between PIRI and each of the MES components; this analysis was performed on the whole group, and sub-groups of males and females. Analyses were performed using the Statistical Package for Social Science (IBM/ SPSS) for Windows (rel. 20.0).

## Results

In the observed population, companies could be classified into two main areas: i) trade and construction; ii) health and social care. The first group comprised 281 workers, the second 290 workers. No difference in mean age was observed between groups (46.3 ± 10.0 vs. 44.7 + 10.3, t = -1.3, p = 0.19), while female gender was more prevalent in health and social care than in trade and construction (81.7% vs. 52%, p<0.001).

The average standardized total PIRI score was 17.5: a very low level which, according to the PIRI Manual [19], corresponds to negligible psychological injury. However, there was a significant dispersion of data, and 139 workers (24.3%) had a score of over 25 that could be interpreted as a potential, definite or significant indication of psychological injury.

An analysis of the lifestyles of our sample of healthy workers revealed that 214 (37.5%) were current smokers, 49 (8.6%) had an alcohol consumption exceeding 1 alcohol unit per die, 388 (68.8%) reported lack of regular physical activity, and 71 (12.4%) reported lack or bad quality of sleep.

Sixty-six workers (11.6%) had high blood pressure, or were taking anti-hypertensive drugs. Central obesity was present in 68 (11.9%) of the sample. 23 workers (4.0%) had reduced HDL cholesterol, 57 (10.0%) showed increased triglyceride level, and 42 (7.4%) had a high glucose level. 182 persons (31.9%) had at least one MES component (Table 1). A diagnosis of MES (3 or more components) was made in 10 subjects (1.7% of the population). No difference in MES prevalence was observed between the two work areas (5 cases each, p = 1.0).

Subjects with a PIRI score of over 25 had a higher prevalence of MES than other workers (4.3% vs. 0.9%); the difference was significant with Fisher exact test (*p* = 0.016). Workers with a high PIRI score also had a higher prevalence of MES components compared to people with low PIRI score: HDL-c reduction (5.0% vs. 3.7%, *p* = not significant); high TGs (15.1% vs. 8.3%, *p* = 0.033); high blood pressure (19.4% vs. 9.0%, *p* = 0.002); high FPG (10.1% vs. 6.5%, *p* = not significant); obesity (12.9% vs. 11.6%, *p* = not significant).

Linear regression analysis showed that the standardized PIRI score was significantly related to the number of MES components (beta = 0.16, *p*<0.001).

The results of logistic regression analysis revealed that psychological injury, detected by a high PIRI score, was significantly related to the presence of at least one MES component, with an odds ratio (95% confidence interval) of 1.78 (1.20–2.64). The association of psychological injury with MES components was still significant when individual factors (gender, age, smoke habit, alcohol intake, and sleep deprivation) were included in the model (Table 2). With the introduction of demographic and lifestyle variables in the logistic model, the coefficient of determination R^2^, a statistic that provides some information about the goodness of fit of a model, significantly increased. Age was significantly associated with MES, while odds for male gender, use of alcohol, lack of physical activity and bad sleep habit increased but failed to reach statistical significance.

**Table 2:** Association between psychological injury (PIRI score >25) and personal factors (age, sex, smoking habit, alcohol use, lack of physical exercise, lack of sleep) and the presence of at least one component of metabolic syndrome (obesity, hypertension, dyslipidemia, diabetes).

	Crude		Adjusted	
	OR (CI95%)	*p*	OR (CI95%)	*p*
High PIRI	1.78 (1.20–2.64)	<0.004	1.60 (1.03–2.48)	<0.05
Female Sex			0.71 (0.48–1.06)	n.s.
Age			1.06 (1.04–1.08)	<0.001
Smoke			0.87 (0.59–1.28)	n.s.
Alcohol			1.23 (0.65–2.33)	n.s.
Lack of Activity			1.06 (0.71–1.58)	n.s.
Sleep deprivation			1.02 (0.58–1.81)	n.s.
R^2^ (coefficient of determination)	0.02		0.11	

Using logistic regression, we also determined whether a high PIRI score was associated with each of the individual MES components (Table 3). We observed increased odds ratios for low HDL cholesterolemia, high fasting glucose, hypertriglyceridemia, and high blood pressure. The association was statistically significant only for the latter two, while for the first two confidence intervals included the hypothesis of equivalence. We failed to observe an increased risk of obesity in individuals with a high PIRI score (Table 3). Separating data by gender revealed a significantly increased OR for arterial hypertension in female workers and a higher OR for hypertriglyceridemia in males. We also observed an increased risk of high cholesterol in male workers, although this association failed to reach a statistically significant level.

**Table 3:** Odds ratios and 95% confidence intervals calculated by logistic regression analysis to identify the adverse outcomes of job strain.

Outcomes	Odds ratio (95% CI)		
	Whole group	Male	Female
Low HDL cholesterolemia	1.34 (0.56–3.42)	3.78 (0.80–17.74)	0.87 (0.27–2.75)
Hypertriglyceridemia	1.96 (1.10–3.14)*	2.53 (1.03–6.22)*	2.08 (0.95–4.56)
High blood pressure	2.43 (1.42–4.14)***	2.51 (0.93–6.76)	2.45 (1.29–4.66)**
High fasting glucose	1.61 (0.83–3.17)	1.89 (0.65–4.99)	1.97 (0.77–5.05)
Increased waist circumference-BMI	1.14 (0.64–2.02)	0.81 (0.22–2.95)	1.22 (0.63–2.35)

* p<0.05;

** p<0.05;

***p>0.001

## Discussion and Conclusions

Our study demonstrated that psychological injury arising from ordinary everyday work situations was significantly associated with MES components. The cross-sectional character of our study prevented us from drawing conclusions about causality. However, these findings are inserted in the flow of literature and support the idea that occupational stress may play a role in the development of MES. A previous study based on the Whitehall II cohort demonstrated a significantly dose-dependent association between self-perceived stress at work and the risk of developing MES [23]. The CARDIA study cohort also showed that workers in high strain jobs had a significantly increased risk of MES compared with those in low strain jobs after 5 years of follow-up. However, the association was only of borderline significance after adjusting for socio-demographic factors, health behaviors and depressive symptoms [14]. The association between psychological injury and MES is also weak in our present study and the percentage of variance explained by logistic regression model is low. This means that, in addition to the factors measured in our study, there are many others that influence the presence of MES.

Our study was conducted on a number of small companies in which workers were engaged in a variety of jobs. Unlike workers who have to ensure public order or provide assistance in case of fire or emergency, the workers from these companies were not exposed to major sources of trauma. Nevertheless many of them reported common traumatic events of working life. The psychological consequences of these injuries can easily be measured and are associated with some dismetabolic components, such as dyslipidemia and hypertension. These associations are influenced by gender. Although the limited number of reports did not always enable us to establish statistical significance, the association trend is clear and corresponds to what is reported in literature. A systematic review of prospective cohort studies on chronic stress and dyslipidemia indicated that there was a potentially positive association, although no definite conclusion could be reached on account of the insufficient number of studies [15]. With regard to dyslipidemia, the results of our study correspond with those observed in the Whitehall II study, where low justice at work was associated with the development of reduced HDL cholesterol and elevated triglycerides among men but not among women [24].

An analysis of the literature indicates that there is a highly complex relationship between stress and hypertension. Previous studies and meta-analytic reviews about the effects of stress on hypertension have often led to conflicting results, or small size effects [25, 26], raising doubt about the extent to which elevations in blood pressure are consistently related to stress. However, despite methodological differences, a recent meta-analysis of case–control and cohort studies of good methodological quality showed positive associations between hypertension and job strain [27]. Since the term "occupational stress" is very broad, it is likely to include various conditions, each of which may or may not have health effects. Different types of work stress have been found to affect blood pressure differently in men and women [15]. Both hormonal and social factors may explain the different response to stress in the two genders. Some studies have obtained results similar to ours; for example, studies on job satisfaction showed that dissatisfied women were more at risk of increased blood pressure after time, whereas job satisfaction failed to have an influence on blood pressure in men [28]. In another study, occupational stress was found to be associated with an increased risk of hypertension in older women, while no association was found in men [29]. However, if a different way of measuring work-related stress is undertaken, results may be different: worrying about keeping one’s job was significantly associated with the development of hypertension in men, but not in women [30]. Job insecurity, in general, is a powerful factor of hypertension, both in studies performed many years ago [31] and in more recent findings [32]. The threat of losing a job, however, is only one of the many possible work-related traumas. If a study adopts, as in our case, a questionnaire that measures the effects of all types of trauma, this inevitably reduces the specificity of the health effects.

We observed a slight, non-significant increase in the risk of type 2 diabetes mellitus; this finding was in line with literature that supports an association, even if the increase in odds is modest [33, 34]. In our sample, among the various components of MES, only obesity was not related to psychological injury. It has, in fact, been reported that the relationship between stress and MES is U-shaped: stress may cause some people to eat less and lose weight and others to eat more [35]. For this reason, there has been some disparity in study findings, even though the majority of research has indicated a relationship between stress and weight gain [15].

Little is known about the nature of the co-occurrence of psychological injury and metabolic syndrome. Individuals suffering from work-related psychological injury may be more prone than the general population to metabolic risk behaviors. In our sample we tested for excessive alcohol use, smoking, low physical exercise, and sleep loss; however, we cannot exclude the presence of other risky behaviors, such as low self-care and excessive caloric intake which may contribute to the increased risk of metabolic diseases. We can however rule out the use of antipsychotic drugs, indicated as a possible cause of the increased incidence of MES in patients with PTSD [36].

The main limitations of this study are due to its cross-sectional design and limited number of observations. We must keep in mind that in populations of active workers, the healthy worker effect reduces the prevalence of the disease and, consequently, the power of the studies. In this study, we calculated an 88.2% statistical power (α = 0.05) for blood pressure, and a 69.6% for MES. This means that 69.6–88.2% of studies with this sample size would be expected to yield a significant "true" effect, when the effect exists. A higher number of observations would protect against the Type II error, i.e. failing to detect an association that is real. Nevertheless our study still shows that it is essential to measure stress at baseline and monitor changes over a number of years, since the burden might fluctuate over time. Occupational physicians who carry out medical surveillance of workers by examining them in the workplace on a yearly basis are in a unique position to collect these data without high costs, and to achieve health promotion intervention at a very low cost/benefit ratio [37].

A review of the literature indicates that not all the tools used to measure work-related stress are equally effective, and this hinders understanding of the relationship between stress and MES [15]. The PIRI questionnaire proved to be an effective means of correlating with MES. This was probably due to the fact that it explicitly takes into account not only the symptoms that appear after trauma, but also some specific complaints such as sleep loss, fatigue and recovery failure that are important in relation to MES.

In conclusion, checking stress levels and monitoring for signs of psychological damage in workers appear to be important measures for preventing MES. Occupational physicians should regularly assess metabolic syndrome in workers suffering from work-related distress and psychological injury. Assessment of medical co-morbidity and lifestyle habits, measurement of weight or waist circumference and blood pressure, and regular blood tests for fasting glucose and lipids should become a key part of the long-term routine monitoring of these workers.
